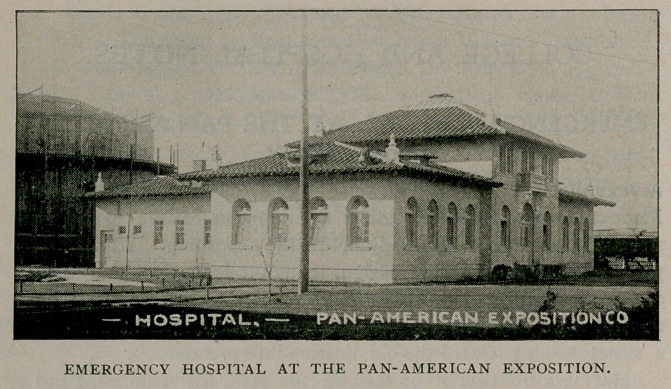# College and Hospital Notes

**Published:** 1901-04

**Authors:** 


### COLLEGE AND HOSPITAL NOTES.

### EMERGENCY HOSPITAL AT THE PAN-AMERICAN EXPOSITION.

A very pretty hospital building stands near the west end of the mall. Floor area rather than elevation is a prominent feature in the construction of this important adjunct to the exposition. Utility is the prime consideration in this design, though it is by no means a case of utility unadorned. In conformity with the general exposition plan the free Spanish renaissance has been treated, in this instance, with a strong leaning toward the old mission interpretation.

Having a frontage of 90 feet on the mall, the main wing has a depth of 38 feet with a height of but one story, except in the center, where it assumes the form of a square tower with a rounded top. This tower attains to the pretentious height of two stories, surmounted with two flagstaffs. One staff supports the exposition flag and the other waves the well known Red Cross banner, the only universal international emblem that is recognised and respected in all countries.

A rear wing, one story high, runs back from the center portion a distance of 56 feet, with a width of 32 feet. This form of construction lends itself readily to this picturesque reminder of the early struggles of our first missionaries.

Color, here as everywhere throughout the grounds, adds its touch of beauty to the odd and in many cases obsolete methods of construction, penetrating rather than clothing the building in the warm changing tints of the sunset. A low wandering adobe mission house, covered with heavy red tiling, its weather stains retouched by the gorgeous rays of the departing sun, may be readily imagined while looking at this rehabilitation of the past.

Any antiquated illusion that may be conveyed by the outside appearance of this building is, however, at once dispelled by a visit to the interior. Modern arrangements that are both convenient and sanitary, mark every feature. Approved medical and surgical appliances have been carefully selected, in regard especially for their adaptability to emergency work and the exigencies that are likely to arise. The main hospital entrance is from the mall, opening directly into a handsome rotunda decorated with tropical plants and suitable hangings of pictures, drapery, and the like.

EMERGENCY HOSPITAL AT THE PAN-AMERICAN EXPOSITION.

The main office is situated at the farther left hand corner of this rotunda, where it is carefully tucked away under the staircase, forming an irregular alcove. It contains telephone and electrical annunciator, messenger call service, together with other modern and necessary appurtenances. As this is lighted from above and encircled by a round gallery opening through the upper story, the effect is very pleasant and agreeable. The first floor front contains in the extreme western wing, two male wards with seven cots each, a bath room, physicians' office, a morgue and a linen chest. The eastern wing contains a woman's ward, large enough to hold a dozen cots, with direct communication to the woman's bath room. This wing also contains an office for the superintendent of nurses, private physician's office, a linen closet and other conveniences.

The upper story is intended for the use of the resident physician and the necessary attendants. It is fitted up with four pleasant, comfortable bed rooms and a bath room. The rear wing, extending back from the main entrance, contains the operating room, sterilising department and instrument cases. Immediately across the hall is the emergency bath room and patients' waiting room. Still farther down the corridor is located the kitchen, pantry and dining room, which is intended for the use of patients only, as the staff have their culinary department in the service building, situated but a few yards distant. In the extreme southern end of this ring is the storage room for the electrical ambulances; this room also contains a station for recharging the batteries, electricity for this purpose being brought from an electric circuit provided for the electric launches on the grand canal. In addition to the two electrical ambulances, a steam or gasoline motor ambulance will be provided to be ready in case of a possible failure of the electrical current. The building is provided with natural gas for heating purposes and for cooking when necessary for the patients.

Water, gas and electricity is carried to every part of the hospital in the most approved manner. The building is plastered throughout and rendered sanitary and germ proof so far as possible, in every portion. The staff in attendance is uniformed as to grade according to universal custom.

In the matter of equipment and appliances, everything is of the newest and best. A new litter attracts considerable attention; it is carefully balanced and so arranged that one attendant can operate it easily and noiselessly as it runs on two wheels about 20 inches in diameter, which are fitted with large inflated rubber tires. Sterilising apparatus with an apartment for instruments and another for towels and linen, is another necessary arrangement.

Dr. Roswell Park is the medical director, Dr. Vertner Kenerson is deputy medical director, and Dr. Alexander Allen is the resident physician,—a staff which will at once indicate medical and surgical skill in the care of patients in this hospital. The efficiency aimed at in this department is an illustration of the manner in which the exposition affairs are administered in all its departments. Everything has been carefully arranged according to a great comprehensive plan, the details of which have been worked out in every instance with careful conscientious precision.

In regard to the importance of this adjunct to the exposition it may be said that up to the 1st of March, 504 cases have been treated on the grounds, only one of which proved fatal. These include all forms of sickness and accidents to workmen employed upon the construction work. In this relation it is well to note that the number of cases treated at the Omaha exposition was about 3,000, while the history of the hospital at the World's Fair in Chicago gives a total of 11,602 medical and surgical cases treated, resulting in 69 deaths.

It is hoped to have less use than this for the hospital at the Pan-American, though in the immense crowds who will attend, no doubt, many individuals will have occasion to appreciate the provision that has been made in this direction.

---

The German Hospital, located on Jefferson Street, near Genesee, Buffalo, N.Y., was opened for patients March 11, 1901. It is one of the finest hospitals in the country, whether considered from the viewpoint of construction, equipment, or hygienic arrangement. It has accommodations for about 72 patients, and both wards and private rooms are models of perfection.

The medical and surgical staff is as follows: President, Charles H. W. Auel; vice-president, Marcel Hartwig; secretary, Charles Weil. Internal medicine—consulting physicians—Conrad Diehl, Emil S. Tobie, Thomas Lothrop; attending physicians, H. C. Buswell, William Meisberger, Julius Ullman, Robert Hebenstreit; surgery—consulting surgeon, Roswell Park; attending surgeons, M. Hartwig, Herman Mynter, J. G. Meidenbauer, Henry G. Bentz; gynecology—consulting physician, Matthew D. Mann; attending physicians, Charles H. W. Auel, Max Breuer, Herman E. Hayd, Sigmund Goldberg; obstetrics—consulting physician, Charles H. W. Auel; diseases of children, L. Schroeter, Charles Weill, H. C. Rooth; eye and ear—consulting physicians, Lucien Howe, Julius Pohlman; attending physician, Jacob Goldberg; nose and throat, attending physicians, G. F. Cott, W. S. Renner; skin—consulting physician, Ernest Wende; attending physicians, Grover Wende, J. Kraus, Alfred E. Diehl; genito-urinary, Alois Jokl, Julian A. Riester; nervous diseases—consulting and attending physicians, W. C. Krauss, Floyd S. Crego, H. G. Matzinger; pathology, W. G. Bissell, H. R. Gaylord, J. A. Miller.

---

The new Consumption Hospital, an adjunct to the Erie County Hospital, now building on the site of the one destroyed by fire about a year ago, is well under way. Ground was broken in February, and the foundation is about completed. The structure will cost about $50,000.

THE Mercy Hospital, of Pittsburg, Pa., has established a department for the treatment of patients bitten by rabid animals. The treatment given is by inoculation, according to the method devised by Pasteur for the prevention of hydrophobia. It is in charge of Dr. A. Letévé, who has had ten years' experience in this work in the Pasteur Institutes in Lille, France, and in New York City. The fee for treatment is one hundred and fifty dollars in advance, including board and lodging.

For additional information, address Pasteur Department, Magee Pathological Institute, Mercy Hospital, Pittsburg Pa.

---

THE regents of the University of the State of New York, at a meeting held March 14, 1901, appointed Drs. Joseph P. Creveling, of Auburn, and Eugene Beach, of Gloversville, as medical examiners representing the Medical Society of the State of New York, to succeed themselves. Drs. Willard N. Bell, of Ogdensburg, and John B. Garrison, of New York, were appointed medical examiners representing the Homeopathic Medical Society of the State of New York. Dr. William Gilman Thompson, of Cornell Medical School, and Dr. Willis G. Tucker, of Albany Medical School, were appointed members of the medical council for five years and three years respectively, in place of Drs. Mann and Didama, who retire.

---

THE Fitch Hospital was closed February 28, 1901. This hospital has been managed by the Buffalo charity organisation society as an accident hospital for several years.

---

THE Manhattan Maternity Hospital and Dispensary, a gift to the poor of the east side of the city of New York by a man who has long been interested in the betterment of conditions in that locality, was incorporated at Albany, March 18, 1901. The incorporators are: Daniel S. Lamont, Cornelius Vanderbilt, Frank L Polk, Henry S. Thompson and William Thorne, of New York City; Moses Taylor, of Mount Kisco, and Percy R. Pyne, of Bernardsville, N.J.

---

THE Batavia Hospital, of which mention has been made heretofore in these columns, appears to have reached a stage of certainty as to its construction. A site has been purchased and plans have been drawn looking to the erection of a two.story building, 61 feet wide and 53 feet deep, at an estimated cost of $6,000. This will leave from the amount raised a goodly sum for equipment. The work thus far accomplished has been done entirely by women.

THE New York Skin and Cancer Hospital has announced the following course of clinical lectures on syphilis, by members of the visiting and consulting staffs, on Wednesday at 4.15 p.m : March 6, Syphilis as a disease; modes of infection; extra-genital syphilis, L. Duncan Bulkley; March 13, Skin manifestations of syphilis, L. Duncan Bulkley; March 20, Infantile syphilis, A. Jacobi; March 27, Syphilis of the mouth, nose, throat and larynx, D. Bryson Delavan; April 3, Syphilis of the eye and ear, David Webster; April 10, Syphilis of the nervous system, Edward D. Fisher; April 17, Syphilis of internal organs, Edward G. Janeway; April 24, Syphilis of the bones, and surgical relations of syphilis, Willy Meyer; May 1, Synopsis, conclusions and treatment of syphilis, L. Duncan Bulkley. The course is free to members of the medical profession on presentation of their professional cards.

## Figures and Tables

**Figure f1:**